# Improving vaccination against seasonal influenza among healthcare workers, 1994-2015

**DOI:** 10.1186/2047-2994-4-S1-P17

**Published:** 2015-06-16

**Authors:** A Iten, C Bonfillon, S Boymond, C-A Siegrist, D Pittet

**Affiliations:** 1 Infection Control Program, University of Geneva Hospitals, Geneva, Switzerland; 2 Hospital Health Service, University of Geneva Hospitals, Geneva, Switzerland; 3 Department of Child and Adolescent Medicine , University of Geneva Hospitals, Geneva, Switzerland

## Introduction

Vaccination against seasonal influenza (SI) is recommended for healthcare workers (HCW) in acute and long-term care facilities. Since vaccination cannot be made mandatory in Switzerland, an alternative was implemented since 2012 at HUG: HCW were obliged to be vaccinated or to wear a mask in ward corridors and patient rooms during SI epidemics. We describe the 20 years’ experience at HUG.

## Objectives

We describe the 20 years’ experience at HUG.

## Methods

From 1994, data are collected annually about vaccinated HCWs at HUG. In addition, regular audits are performed to assess compliance with recommendations. Since 2012, during annual SI epidemics, audits recorded compliance of HCWs with a badge meaning “Vaccinated” and those with correct use of a mask over several 2 week-periods. Compliance was assessed as follows: (number of HCWs wearing a colored badge + number of HCWs wearing a mask correctly)/number of HCWs observed = number of compliant HCWs/number of HCWs observed, expressed as a percentage.

## Results

In 1994, 14% of HCWs were vaccinated against SI. Following an investigation, information campaigns, lectures about influenza and the influenza vaccine, and on-site vaccination were offered from 1995 to 2008. During this period, the proportion of vaccinated HCWs’ varied between 21% and 27%. In 2009, HCWs had to choose between vaccination attested with a badge or correct mask wear. In 2009, the year of H1N1 pandemic, 49% of HCWs were vaccinated, in 2010 27%, in 2011 29%.

Since winter 2012, HCWs vaccinated against SI wear a badge with the text "I am vaccinated to protect you" and those who were not vaccinated wear a badge with the text "I wear a mask to protect you". Of 1390 HCWs observed in winter 2012, 469 wore a badge or mask (estimated compliance, 33.5%). In winter 2013, 2070/2937 observed HCWs were compliant (70.5%), in winter 2014, 3516 /4459 observed HCW (70.5%). Data collection is in progress for winter 2015. In parallel, we observed an increase of vaccinated HCW: 46.6% in winter 2015.

## Conclusion

Offering the choice between vaccination and mask wear, associated with mandatory badge wear and assessment of mask compliance respectively, can be an effective approach to increase HCW vaccination rate against SI.

## Disclosure of interest

None declared.

